# Investigation and Modeling of the Behavior of Temperature Characteristics of 0.3–1.1 GHz Complementary Metal Oxide Semiconductor Class-A Broadband Power Amplifiers

**DOI:** 10.3390/mi15020246

**Published:** 2024-02-06

**Authors:** Ruiliang Li, Shaohua Zhou, Cheng Yang, Jian Wang

**Affiliations:** 1 Qingdao Institute for Marine Technology of Tianjin University, Qingdao 266200, China; tjliruiliang@163.com (R.L.); zhoushaohua@tju.edu.cn (S.Z.); 2 ZJU-Hangzhou Global Scientific and Technological Innovation Center, Zhejiang University, Hangzhou 310058, China; 3 School of Microelectronics, Tianjin University, Tianjin 300072, China; 4 Shandong Engineering Technology Research Center of Marine Information Perception and Transmission, Qingdao 266200, China

**Keywords:** PA, temperature, SVM, model, CMOS, class-A

## Abstract

A power amplifier (PA) stands as a central module within the electronic information system (EIS), and any variation in a PA’s specifications has a direct impact on the EIS’s performance, especially in the face of temperature fluctuations. In examining the influence of PA specification changes on the EIS, we employed support vector machine (SVM) to model the behavior of the temperature characteristics of 0.3–1.1 GHz complementary metal oxide semiconductor (CMOS) class-A broadband PAs. The results show that the parameters of S_11_, S_12_, S_21_, and S_22_ can be effectively modeled. SVM outperforms Elman and GRNN in terms of combined modeling time and modeling accuracy. This research can be extended to modeling the behavior of other types of power amplifiers or devices and circuits.

## 1. Introduction

Modern wireless communications have revolutionized our lives, and they are also becoming one of the critical supporting technologies in several areas of industry [1]. Power amplifiers (PAs) are the most critical element of modern wireless communication systems and significantly impact the performance of wireless communication systems [2]. PAs have a significant temperature dependence [3,4]. The degradation of the PA specification can lead to accelerated system failures if care is not taken to understand the effects of temperature changes on the PA [5,6]. Therefore, to avoid the system’s failure, the temperature characteristics of the PA need to be considered in advance during the optimization of the system design. To understand the temperature characteristics of a PA, there is a need to model the relationship between temperature variation and PA specifications.

Support vector machine (SVM) is a machine learning algorithm which was proposed by V. Vapnik et al. in the 1960s [7,8]. SVM can be used to describe the relationship between different variables. It is widely used in many fields as it minimizes real risk by seeking to minimize structural risk and pursues optimal results under limited information conditions [7,9]. For example, Chen et al. proposed a privacy-preserving medical diagnosis scheme based on a multi-class SVM [10]. The scheme is based on the Distributed Double Trapdoor Public Key Cryptosystem (DT-PKC) and the Boneh–Goh–Nissim (BGN) cryptosystem. Chen et al.’s scheme can deal with both linearly divisible data and nonlinear data, while preserving the privacy of the user’s data and support vectors. Chhavi Gupta et al. used a supervised machine learning (ML) approach to create a predictive model for the degree of work–life balance (on a scale of 1–5) of female professionals in information technology (IT) [11]. The results of the study showed that the optimized SVM model had the highest accuracy in work–life balance prediction. In addition, Chhavi Gupta et al. compared the predictive ability of the SVM model with that of the multiple regression model. A comparison of these two prediction models revealed that SVM was more accurate than multiple regression. Liu et al. proposed a self-induction method using a hybrid kernel function fuzzy support vector machine (FSVM) displacement prediction model to solve the problems of the large size, high cost, and low reliability of the displacement sensor in the magnetic levitation bearing system [12]. The simulation results show that the performance of the prediction model using the hybrid kernel function FSVM is significantly better than that of the prediction model using other kernel functions, and the predicted values in the x-direction and y-direction are about 93.35% and 95.5% of the actual values, respectively. Amyloid-based diagnostic biomarkers and assays provide only limited information about the disease process, and they are unable to identify diseased individuals before amyloid-β accumulates in the brain in large quantities. Chima et al. developed a method for early AD detection based on an SVM to identify potential blood-based, non-amyloid biomarkers [13]. Lu et al. has optimized the adhesive stamp mass transfer process for micro light-emitting diodes (micro-LEDs) using a support vector machine (SVM) model [14]. And from them, the separation speed and force between the stamp and the donor substrate were extracted as signal features. Finally, the classification accuracy based on the SVM model reached 85%, which implies that a more complex definition of the signal features is needed in future work. During the operation of an electric locomotive, a large amount of operational data will be stored by the analog-to-digital converter (ADC) and recording oscilloscope of the on-board traction control unit (TCU). It is of great significance to analyze data quality to detect slippage for energy saving, performance improvement, and safety of the traction control system of electric locomotives. Huang et al. proposed an SVM slippage detection method based on the EWT and FE algorithms after analyzing and summarizing the advantages of the Empirical Wavelet Transform (EWT) and the Fuzzy Entropy (FE) algorithms in data processing, as well as the classification theory of the support vector machines (SVMs) algorithm for SVM slip detection [15]. Brazil et al. developed an advanced method to classify load–pull contours for broadband high-efficiency PA designs using the technique of SVM [16]. This method achieves high efficiency and linearity over the entire operating bandwidth. Shi et al. proposed a model for PAs by adding a decomposed vector rotation (DVR) basis term to a support vector regression (SVR) model based on real-valued time delays [17]. This model achieves more than a 2 dB improvement in normalized mean square error (NMSE) and has better stability. Geng et al. proposed a PA model based on amplitude and phase-augmented time-delayed dual SVR [18]. The proposed model reduces CPU training time and provides efficient linearization performance with improved generalization capability. In summary, SVM has shown good application prospects and achieved remarkable results in many fields such as biology, medicine, electrical machinery, information statistics, device modeling, and optimization. Therefore, the emergence of SVM provides a new idea and opens up a new way to construct a nonlinear relationship between the specification of radio frequency (RF) power amplifiers and temperature change.

In this paper, the authors applied SVM for modeling based on the measured data of 0.3–1.1 GHz CMOS PAs at three different ambient temperatures (−40 °C, 25 °C, 125 °C). The experimental results show that the SVM can better describe the relationship between the S-parameters of the PA and the temperature variation. Meanwhile, the authors also compared the modeling results of SVM with those of Elman and Generalized Regression Neural Network (GRNN). The comparison results show that SVM outperforms Elman and GRNN under the combined consideration of modeling time and modeling accuracy.

The remainder of this paper is structured as follows: Section 2 introduces the methods; Section 3 explains the measurement environment and measured data for modeling; Section 4 shows and discusses the modeling results; and Section 5 summarizes the work conducted in this paper.

## 2. Methodology

The SVM concept was first proposed in 1964 and then gradually theorized with the progress of pattern recognition and became a part of statistical learning theory [8]. It developed rapidly and derived a series of improved and extended algorithms in recent decades. Except for modeling PA behavior, this method is widely used in mortality prediction due to COVID-19 [19], coronary artery disease detection [20], wireless communication channel scenarios identification [21], image classification [22], power quality disturbance classification [23], etc. The regression is revealed as a branch of SVM. By creating a “margin” besides the linear function, the SVM model skips calculating the loss for these samples falling into it. The filtered support vector will impact its function and obtain the optimized model by maximizing the margin correlating to the loss function.

The SVM employs the principle of structural risk minimization, enabling the better avoidance of local optima and exhibiting lower generalization error. By utilizing a kernel function, SVM maps nonlinear problems from low-dimensional space to high-dimensional space, facilitating effective linear separation in the higher-dimensional realm and addressing complex nonlinear issues. Moreover, SVM demonstrates robustness against noise and outliers in the training data, efficiently handling noise present in input data. As a supervised learning algorithm, SVM exhibits excellent generalization performance in the training and learning of small-scale samples. Considering the scale of the data in this paper, SVM is chosen as the primary modeling method.

Here, we investigate the effect of SVM models on the behavioral modeling of the temperature characteristics of CMOS class-A broadband PAs, and the experimental data of a 0.3–1.1 GHz broadband PA are used as a modeling dataset. For the given training datasets {(*f*_1_, *T*_1_, S_111_, S_121_, S_211_, S_221_), (*f*_2_, *T*_2_, S11_2_, S12_2_, S21_2_, S22_2_), …, (*f*_m_, *T*_m_, S_11m_, S_12m_, S_21m_, S_22m_)}, a regression SVM model *f*(*x*) = *c*∙*x*+*d* is hoped to get as close to *y_i_* as possible, where x*_i_* = (*f*_i_, *T*_i_), *y_i_* = (S_11i_, S_12i_, S_21i_, S_22i_), *f*_1_ is the frequency, and *T*_1_ is the temperature. The weight factor *c* and the threshold value *d* will be determined during the process. Considering a deviation of *ε* between *f*(*x*) and *y_i_* in our required limit, the loss is calculated only if the absolute difference is greater than *ε.* The SVM model can be formalized as:

(1) $$\underset{w,d}{\min}\frac{1}{2}||c|{|}^{2}+R\sum_{i=1}^{m}l_{\varepsilon }(G(f_{i},T_{i})),S_{11i},S_{12i},S_{21i},S_{22i})$$

where *R* is the regularization constant, and *l* is the *ε*-insensitive loss function shown in Equation (2):

(2) $$l_{\varepsilon }(z)=\left\{\begin{array}{ll} 0, & \;|z|\leq \varepsilon \\ |z|-\varepsilon , & \mathrm{otherwise} \end{array}\right.$$

Equation (1) can be rewritten by introducing the slack variables $\zeta_{i}$ and ${\hat{\zeta }}_{i}$:

(3) $$\begin{array}{c} \underset{c,d,\zeta_{i},{\hat{\zeta }}_{i}}{\min}\frac{1}{2}||c|{|}^{2}+R\sum_{i=1}^{m}\left(\zeta_{i},{\hat{\zeta }}_{i}\right)\\ \mathrm{s.t.}\\ G(x_{i})-y_{i}\leq \varepsilon +\zeta_{i},y_{i}-G(x_{i})\leq \varepsilon +{\hat{\zeta }}_{i},\zeta_{i}\geq 0,{\hat{\zeta }}_{i}\geq 0,i=1,2,\ldots ,m. \end{array}$$

The loss function is transformed into a simpler dual calculation by introducing Lagrange multipliers.

(4) $$\begin{array}{l} L(c,d,\beta ,\hat{\beta },\zeta ,\hat{\zeta },\eta ,\hat{\eta })\\ \;=\frac{1}{2}||c|{|}^{2}+R\sum_{i=1}^{m}\left(\zeta_{i}+{\hat{\zeta }}_{i}\right)-\sum_{i=1}^{m}\zeta_{i}\eta_{i}-\sum_{i=1}^{m}{\hat{\zeta }}_{i}{\hat{\eta }}_{i}\\ \;+\sum_{i=1}^{m}\beta_{i}(G(x_{i})-y_{i}-\varepsilon -\zeta_{i})+\sum_{i=1}^{m}{\hat{\beta }}_{i}(y_{i}-G(x_{i})-\varepsilon -{\hat{\zeta }}_{i}) \end{array}$$

where *L* is the Lagrange function, and $\eta_{i}$, ${\hat{\eta }}_{i}$, $\beta_{i}$, and ${\hat{\beta }}_{i}$ are the Lagrange multipliers.

Assuming that the partial derivative of $L(c,d,\beta ,\hat{\beta },\zeta ,\hat{\zeta },\eta ,\hat{\eta })$ with respect to *c*, *d*, $\zeta_{i}$, and ${\hat{\zeta }}_{i}$ is set to zero, we can obtain:

(5) $$c=\sum_{i=1}^{m}({\hat{\beta }}_{i}-\beta_{i})x_{i},$$

Karush–Kuhn–Tucker (KKT) conditions should be met in the above process, namely

(6) $$\left\{\begin{array}{c} \beta_{i}(G(x_{i})-y_{i}-\varepsilon -\zeta_{i})=0\\ {\hat{\beta }}_{i}(y_{i}-G(x_{i})-\varepsilon -{\hat{\zeta }}_{i})=0\\ \beta_{i}{\hat{\beta }}_{i}=0\\ \zeta_{i}{\hat{\zeta }}_{i}=0\\ (R-\beta_{i})\zeta_{i}=0\\ (R-{\hat{\beta }}_{i}){\hat{\zeta }}_{i}=0 \end{array}\right.$$

The solution of the SVM model is shown in Equation (7).

(7) $$G(x)=\sum_{i=1}^{m}({\hat{\beta }}_{i}-\beta_{i})x_{i}^{T}x+d$$

A robust approach is often adopted by averaging selected multiple samples that satisfy the condition 0 < *α_i_* < *R* that can solve *d*. Considering the feature mapping form, then:

(8) $$c=\sum_{i=1}^{m}\left({\hat{\beta }}_{i}-\beta_{i})\phi (x\right)$$

where *ϕ*(*x_j_*) is the kernel function.

The SVM model can be expressed as:

(9) $$G(x)=\sum_{i=1}^{m}({\hat{\beta }}_{i}-\beta_{i})\kappa (x_{i}^{T}x)+d$$

where *κ*(*x_i_^T^x*) = *ϕ*(*x_i_*)*^T^*.

Compared with the general linear regression model, the SVM model creates a margin only if the differences between *G*(*f*_i_, *T*_i_) and (S11_i_, S12_i_, S21_i_, S22_i_) are greater than *ε.* General linear regression optimizes the model by averaging after gradient descent, while SVM optimizes the model by maximizing the width of the margin and minimizing the total loss. Moreover, this model may be utilized as a powerful tool for modeling the temperature characteristics of PAs based on the feature of continuously approximating the nonlinear function.

## 3. Measurements

**Figure 1:**
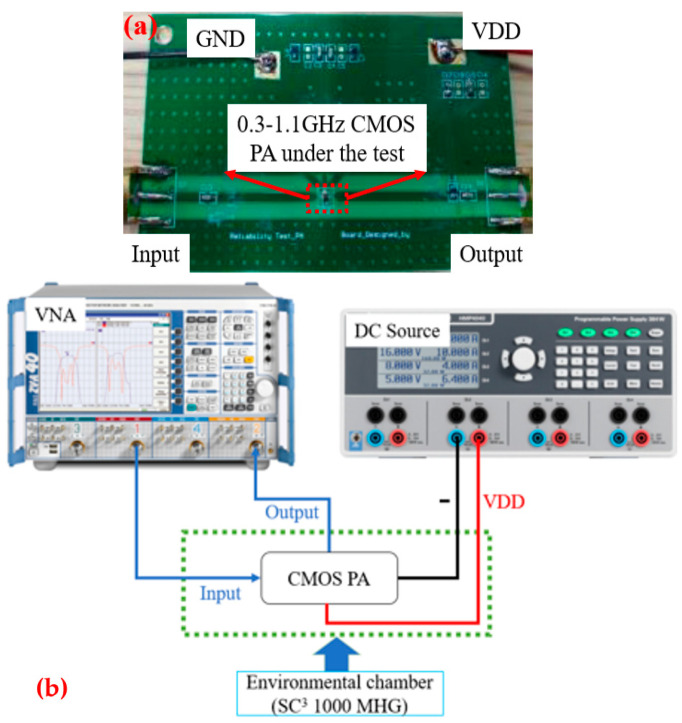
Measurement environment: (**a**) PA to be measured; (**b**) measurement connection diagram.

To investigate the effect of SVM on modeling for the temperature characteristics of CMOS class-A broadband PAs, the temperature characteristics of a 0.3–1.1 GHz broadband PA were measured. To obtain data for SVM modeling, the temperature characteristics of this PA were measured in an experimental environmental chamber in this paper, and the measurement environment is shown in Figure 1. A DC power supply from R&S and a vector network analyzer (VNA) were used during the measurements. The DC power supply provided the required DC bias for the PA, and the VNA was used for the S-parameter measurements and data acquisition.

This paper investigates the effect of SVM models on the behavioral modeling of the temperature characteristics of CMOS class-A broadband PAs. The experimental data of a 0.3–1.1 GHz broadband PA are used as a modeling dataset. According to the input SVM network, the input matrix is defined as

(10) $$X=\left[\begin{array}{cc} f_{1} & T_{1}\\ \cdots & \cdots \\ f_{m} & T_{m} \end{array}\right]$$

where *f*_1_ is the frequency and *T*_1_ is the temperature.

The output data are the characteristics of S_11_, S_12_, S_21_, and S_22_, which can be expressed as

(11) $$T=\left[\begin{array}{c} S_{111}{\;S}_{121}{\;S}_{211}{\;S}_{221}\\ \cdots \\ S_{11m}{\;S}_{12m}{\;S}_{21m}{\;S}_{22m} \end{array}\right]$$

It should be noted that only ¾ (75%) of the measurements are used for modeling, and this 75% is divided into training data (1/4, 25%) and validation data (1/2, 50%).

## 4. Results

### 4.1. S_11_

**Figure 2:**
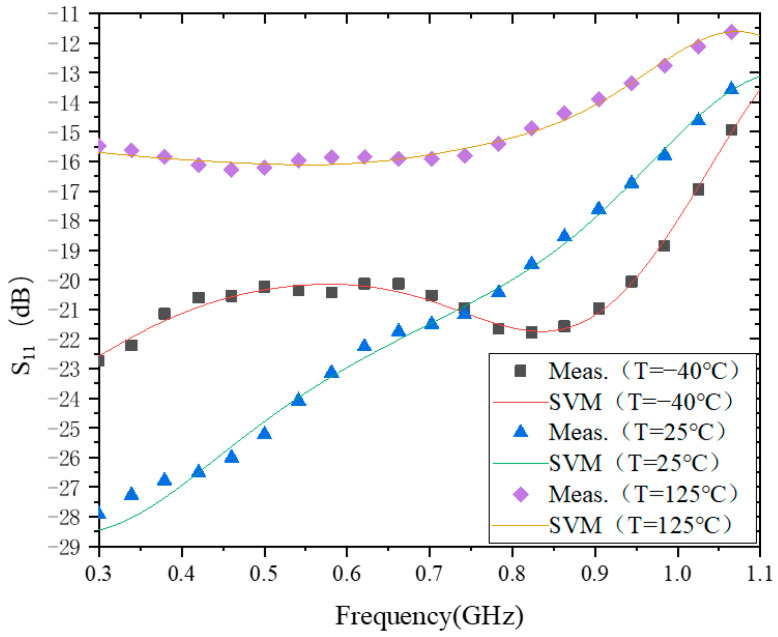
The results of modeling S_11_.

Figure 2 shows the modeling behavior of S_11_ at −40 °C, 25 °C, and 125 °C. The training time of the model is about 13 ms at all three temperatures, and the test errors of the model are 7.5948 × 10^−2^, 1.4313 × 10^−1^, and 2.3117 × 10^−2^, respectively. This indicates that for the behavioral modeling of the temperature characteristics of S_11_, the model’s accuracy can reach the order of 10^−1^ for all of them. However, in terms of model accuracy, the model accuracy of S_11_ at −40 °C and 125 °C is an order of magnitude higher than that at 25 °C. One possible reason for this is that the difference between the maximum and minimum values of S_11_ at −40 °C and 125 °C is smaller than that at 25 °C.

In the 0.3–0.75 GHz range, |S_11_| does not vary monotonically with temperature. For example, from −40 °C to 25 °C, |S_11_| increases with temperature, while from 25 °C to 125 °C, |S_11_| decreases with temperature. In the range of 0.75 to 1.1 GHz, |S_11_| decreases with increasing temperature. The main reason for this phenomenon is that during the design process of the PA, the transistors and matching circuits are designed to consider impedance matching at 25 °C only. When the temperature changes, especially at low temperatures, the carrier mobility in the transistor increases with decreasing temperature, making the impedance of the transistor deviate far from the design value at 25 °C, which results in the variation in the characteristics of |S_11_| at −40 °C over the entire operating frequency range, though this is not quite the same as those at 25 °C and 125 °C.

### 4.2. S_12_

**Figure 3:**
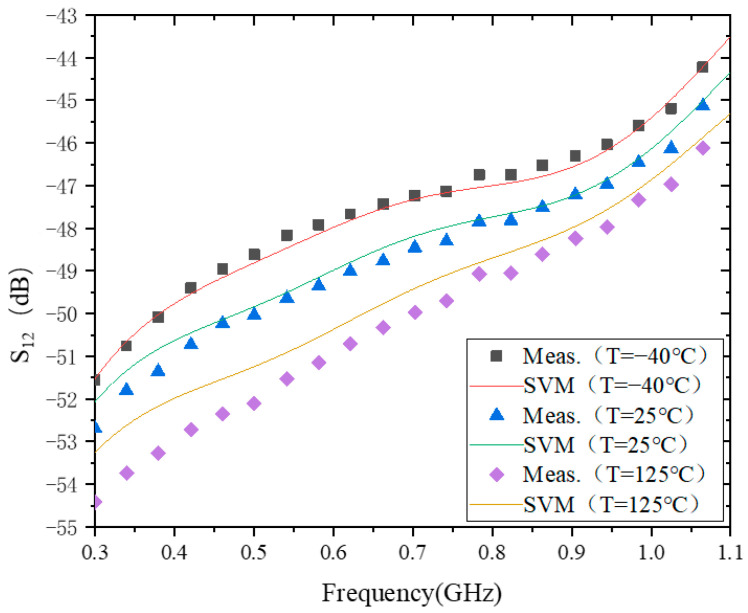
The results of modeling of S_12_.

Figure 3 shows the modeling behavior of S_12_ at −40 °C, 25 °C, and 125 °C. The model accuracy of S_12_ at these three temperatures is 4.3359 × 10^−1^, 4.4285 × 10^−1^, and 1.6438, respectively. From this, the model accuracy at the two temperatures of −40 °C and 25 °C is one magnitude higher than that at 125 °C. One possible reason for this situation is that the difference between the maximum and minimum values of S_12_ at 125 °C is greater than the difference between the two temperatures of −40 °C and 25 °C. According to the model results of S_11_ and S_12_, if the difference between the maximum and minimum values of S_11_ or S_12_ is larger, then the corresponding model accuracy error will also be larger.

In addition, it can be seen from Figure 3 that |S_12_| increases with temperature in the frequency range of 0.3–1.1 GHz. This is different from the characteristic of |S_11_| with temperature. Because |S_12_| and |S_11_| are not defined in the same way, when the temperature changes, it does not necessarily produce the same phenomena as |S_11_| at −40 °C. However, the impedance of the transistors and matching circuits are designed for 25 °C. Therefore, when the temperature deviates from 25 °C, the value of |S_12_| will also deviate from the original design value.

### 4.3. S_21_

**Figure 4:**
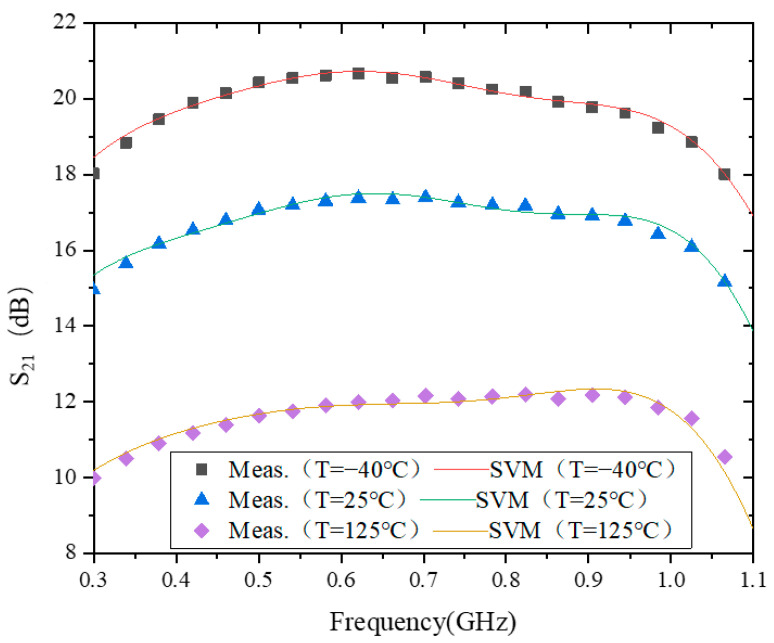
The results of modeling S_21_.

Figure 4 shows the modeling behavior of S_21_ at −40 °C, 25 °C, and 125 °C. The test errors of the model at these three temperatures are 1.9177 × 10^−1^, 2.1655 × 10^−1^, and 4.2388 × 10^−1^, respectively. From this, the model accuracy of S_21_ reaches 10^−1^ at all three temperatures. The difference between the maximum and minimum values of S_21_ is relatively small at all three temperatures. Therefore, there is no case of high errors at individual temperature points as in S_11_ and S_12_. In addition, from a time perspective, the training time of the model is about 13 ms at all three temperatures. This is the same as the case of S_11_ and S_12_.

As can be seen in Figure 4, |S_21_| decreases with increasing temperature. The main reason for this phenomenon is that the carrier mobility in the transistor decreases with increasing temperature. This is analyzed as follows.

Transduction in the unsaturated regions [24,25].

(12) $$g_{mL}=\frac{\partial I_{D}}{\partial V_{GS}}=\frac{W\mu_{n}C_{ox}}{L}V_{DS},$$

And the carrier mobility versus temperature can be expressed as [26].

(13) $$\mu_{n}(T)=\mu_{n}(T_{0}){\left(\frac{T}{T_{0}}\right)}^{-3/2}$$

where *W* is the gate width, *μ_n_* is the carrier mobility, *C_ox_* is the gate oxide capacitance per unit area, *L* is the gate length, *V_GS_* is the gate voltage, *V_T_* is the threshold voltage, and *V_DS_* is the drain voltage, T_0_ = 300 K.

According to Equations (12) and (13), the transconductance of the transistor decreases with increasing temperature. And the transconductance is usually considered to be the gain of the transistor [25]. Thus, it can be observed that the S_21_ of the PA degrades with increasing temperature.

### 4.4. S_22_

**Figure 5:**
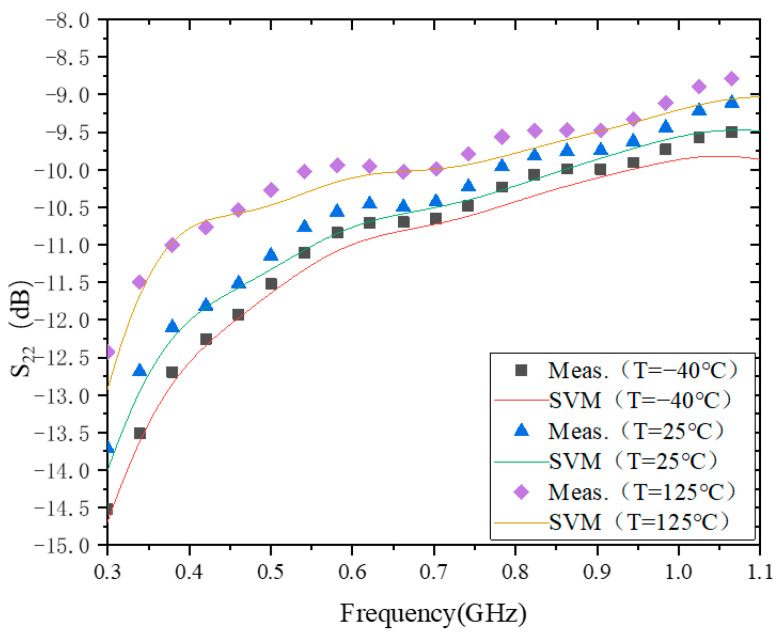
The results of modeling S_22_.

Figure 5 shows the modeling behavior of S_22_ at −40 °C, 25 °C, and 125 °C. The validation errors of the model at three different ambient temperatures are 1.8112 × 10^−1^, 1.9015 × 10^−1^, and 2.2803 × 10^−1^, respectively. From the time perspective, the time required for model training at all three temperatures is about 13 ms.

In addition, it can be seen that |S_22_| decreases with increasing temperature. This is the same as the temperature-dependent characteristic of |S_21_|, but the exact opposite of the temperature-dependent characteristic of |S_12_|. However, the main reason for the change in |S_22_| with temperature is also due to the fact that the impedance matching at 25 °C was only considered during the design process. When the temperature changes, the impedance will also change, which is reflected by the fact that each S-parameter will deviate from the original design reference value. The specific trend of S_11_, S_12_, and S_22_ with temperature changes needs to be specifically analyzed and discussed in conjunction with their respective definitions, and it is also more complicated. The topic of this paper is to use SVM to model the measured data of RF PAs at different ambient temperatures, so we will not discuss this again. In the future, the authors will also specifically address this issue and report on the specific degradation trends and influencing factors.

To better evaluate the performance of the SVM method in modeling, we introduced Elman [27] and the Generalized Regression Neural Network (GRNN) [28] methods that are commonly used for PA modeling for comparison. The results are presented in Table 1. From Table 1, it can be seen that SVM has the shortest modeling time and Elman has the longest modeling time. The modeling accuracy of SVM is higher than Elman and GRNN.

Table 1 shows that the SVM method performs well in the training and learning of small-scale samples, showcasing good generalization performance.

## 5. Conclusions

In this paper, SVM is applied to model the S-parameters of 0.3–1.1 GHz CMOS with measured data at different ambient temperatures. The modeling results show that SVM has a high accuracy and fast modeling speed compared with Elman and GRNN. The results of this paper show that SVM also shows good application prospects and achieves good modeling results in the field of RF PA modeling.

## Figures and Tables

**Table 1:** Modeling results of 0.3–1.1 GHz CMOS class-A broadband PAs.

Specification	0.3–1.1 GHz CMOS PA									
	Temperature	Training Time (ms)			Training Error (MSE)			Test Error (MSE)		
		SVM	Elman	GRNN	SVM	Elman	GRNN	SVM	Elman	GRNN
S_11_	−40 °C	13.1828	22.0649	14.5207	7.6094 × 10^−2^	1.3545 × 10^−1^	8.7519 × 10^−1^	7.5948 × 10^−2^	1.3329 × 10^−1^	8.1818 × 10^−1^
	25 °C	13.1884	21.7823	14.4215	1.4338 × 10^−1^	1.5737 × 10^−1^	5.0432 × 10^−1^	1.4313 × 10^−1^	1.5638 × 10^−1^	4.9011 × 10^−1^
	125 °C	13.3785	21.8585	14.4760	2.3635 × 10^−2^	2.3901 × 10^−2^	1.2462 × 10^−1^	2.3117 × 10^−2^	2.3299 × 10^−2^	1.2311 × 10^−1^
S_12_	−40 °C	13.0228	21.9445	14.2185	4.4906 × 10^−1^	1.2736	5.8912 × 10^−1^	4.3359 × 10^−1^	1.2522	5.6253 × 10^−1^
	25 °C	13.1254	24.2897	13.2567	4.4344 × 10^−1^	5.3769 × 10^−1^	5.9591 × 10^−1^	4.4285 × 10^−1^	5.3987 × 10^−1^	5.7790 × 10^−1^
	125 °C	13.1286	21.9958	13.5437	1.8640	1.9003	2.3674	1.6438	1.8353	2.9581
S_21_	−40 °C	13.2321	21.7983	13.3256	1.9315 × 10^−1^	4.7687 × 10^−1^	3.8398 × 10^−1^	1.9177 × 10^−1^	4.5078 × 10^−1^	3.5495 × 10^−1^
	25 °C	13.3907	21.9360	13.4565	2.1798 × 10^−1^	3.0310 × 10^−1^	3.3669 × 10^−1^	2.1655 × 10^−1^	2.7566 × 10^−1^	3.0555 × 10^−1^
	125 °C	13.335	21.8518	13.769	4.2989 × 10^−1^	4.5583 × 10^−1^	4.7763 × 10^−1^	4.2388 × 10^−1^	4.6239 × 10^−1^	4.4368 × 10^−1^
S_22_	−40 °C	13.1499	30.5666	15.0396	1.8651 × 10^−1^	4.2107 × 10^−1^	3.3053 × 10^−1^	1.8112 × 10^−1^	4.1284 × 10^−1^	3.2324 × 10^−1^
	25 °C	13.3284	25.0988	14.0633	1.9128 × 10^−1^	4.9093 × 10^−1^	2.4705 × 10^−1^	1.9015 × 10^−1^	4.8292 × 10^−1^	2.4147 × 10^−1^
	125 °C	13.1787	23.5633	13.9547	2.2977 × 10^−1^	3.2298 × 10^−1^	2.5628 × 10^−1^	2.2803 × 10^−1^	3.1423 × 10^−1^	2.5102 × 10^−1^

## Data Availability

The data that support the findings of this study are available from the corresponding author upon reasonable request.
